# Parasites and vectors of malaria on Rusinga Island, Western Kenya

**DOI:** 10.1186/s13071-015-0860-z

**Published:** 2015-04-28

**Authors:** Evelyn A Olanga, Lawrence Okombo, Lucy W Irungu, Wolfgang R Mukabana

**Affiliations:** International Centre of Insect Physiology and Ecology, P.O. Box 30772 GPO, Nairobi, Kenya; School of Biological Sciences, University of Nairobi, P.O. Box 30197 GPO, Nairobi, Kenya

**Keywords:** Malaria, Malaria prevalence, *Plasmodium falciparum*, *P. malariae*, *P. ovale*, Malaria, *Anopheles*, *Culex*, *Mansonia*, Indoor transmission, Outdoor transmission, Capture fishing, Rusinga Island, Kenya

## Abstract

**Background:**

There is a dearth of information on malaria endemicity in the islands of Lake Victoria in western Kenya. In this study malaria prevalence and *Plasmodium* sporozoite rates on Rusinga Island were investigated. The contribution of different *Anopheles* species to indoor and outdoor transmission of malaria was also determined.

**Methods:**

Active case detection through microscopy was used to diagnose malaria in a 10% random sample of the human population on Rusinga Island and a longitudinal entomological survey conducted in Gunda village in 2012. Nocturnally active host-seeking mosquitoes were captured indoors and outdoors using odour-baited traps. *Anopheles* species were tested for the presence of *Plasmodium* parasites using an enzyme linked immunosorbent assay. All data were analyzed using generalized linear models.

**Results:**

Single infections of *Plasmodium falciparum* (88.1%)*, P. malariae* (3.96%) and *P. ovale* (0.79%) as well as multiple infections (7.14%) of these parasites were found on Rusinga Island. The overall malaria prevalence was 10.9%. The risk of contracting malaria was higher among dwellers of Rusinga West than Rusinga East locations (Odds Ratio [OR] = 1.5, 95% Confidence Interval [CI] 1.14 – 1.97, P = 0.003). Parasite positivity was significantly associated with individuals who did not use malaria protective measures (OR = 2.65, 95% CI 1.76 – 3.91, p < 0.001). A total of 1,684 mosquitoes, including 74 anophelines, were captured. Unlike *Culex* species, more of which were collected indoors than outdoors (P < 0.001), the females of *An. gambiae* s.l. (P = 0.477), *An. funestus* s.l. (P = 0.153) and *Mansonia* species captured indoors versus outdoors were not different. The 46 *An. gambiae* s.l. collected were mainly *An. arabiensis* (92.3%). Of the 62 malaria mosquitoes tested, 4, including 2 indoor and 2 outdoor-collected individuals had *Plasmodium*.

**Conclusion:**

The rather significant and unexpected contribution of *P. malariae* and *P. ovale* to the overall malaria prevalence on Rusinga Island underscores the epidemiological importance of these species in the big push towards eliminating malaria. Although current entomological interventions mainly target indoor environments, additional strategies should be considered to prevent outdoor transmission of malaria.

## Background

Malaria is a public health problem in Kenya despite intense deployment of vector control tools. Malaria prevalence is highest in areas around the shores of Lake Victoria in western Kenya [1,2]. The main tools used for vector control in this region include long lasting insecticidal nets (LLINs) and indoor residual spraying (IRS) [3]. *Anopheles gambiae* s.l. has been reported to be the main malaria vector in western Kenya [3-6]. The abundance and density of this vector has been clearly documented in the islands of Lake Victoria [4]. However, there is a dearth of information on the role of anophelines in indoor and outdoor transmission in the aforementioned islands. In addition, little information on malaria parasites in these islands is available. Compared to other areas in western Kenya where epidemiological studies have been conducted [5], very little is known on malaria transmission in the islands of Lake Victoria. This study sought to determine malaria prevalence, *Plasmodium* sporozoite rates and the contribution of different *Anopheles* species to indoor and outdoor malaria transmission on Rusinga Island, western Kenya. The association between morbidity and use of malaria preventive measures was also evaluated.

## Methods

### Study site

This study was conducted on Rusinga Island, which is located in Mbita sub-county in western Kenya. Rusinga Island is predominantly rural and is connected to the mainland via a causeway at Mbita Point Township. Residents of Rusinga Island rely on the neighbouring Mbita town for regional transportation, access to banking services and other urban economic services. The island covers an area of 42 km^2^ and the terrain is mainly rocky and hilly. The centre of the Island is characterized by a large hill called Ligogo. Shrubs constitute the main vegetation on the island but the land has been extensively deforested. The island is divided into two administrative sections namely Rusinga East and Rusinga West sub-locations. The dominant ethnic group is *Luo* who mainly engage in artisanal capture fishing and small-scale trading [7]. Subsistence farming of drought-resistant crops is also practised. The subsistence crops cultivated on Rusinga Island include maize, vegetables, potatoes and millet. Malaria in Mbita sub-county is perennial with peaks occurring in July, shortly after the long rainy season [8]. Fishing is the main economic activity on the island and is mainly conducted by adult males. Women are mostly involved in fish processing and trading. Other fishing-related activities that are carried out on the island include boat making and fish net repair.

### Malaria burden and parasite diversity on Rusinga Island

A cross-sectional malaria survey was conducted in all villages on Rusinga Island in October and November 2012 to determine malaria parasite diversity and prevalence. A sample size of 2,240 study participants was determined using the formula proposed by Daniel [9] and Naing *et al*. [10]. The selected study participants were mobilized by project staff performing home visits and requesting human research subjects to turn up at the nearest one of eight primary schools that served as sentinel malaria testing sites.

Each participant’s body temperature was measured using an electronic thermometer (Hangzhou Hua’an Medical & health instruments Co. Ltd., China). Fever was defined as temperature above 37.5°C. Malaria infection status was tested microscopically using participants’ finger pricked blood smears stained with 10% Giemsa. The blood smears were examined by two experienced microscopists. The first one recorded malaria positivity and identified malaria parasites to species level. The second microscopist performed quality assurance before a final result was determined. A slide was categorized as negative if no malaria parasite was seen after scanning 100 microscope fields. Individuals found positive for malaria were treated with a dose of artemether-lumefantrine (AL) according to the Kenya national guidelines for the diagnosis, treatment and prevention of malaria [11]. Malaria prevalence was calculated as the proportion of participants with malaria from the total number of individuals tested.

Gametocyte prevalence was measured to determine the level of human to mosquito malaria transmission potential. The study participants were screened for gametocytes of *P. falciparum* only. Blood smears were considered negative if no gametocyte was detected after examining 100 fields. Gametocyte prevalence was calculated as the proportion of human subjects harbouring gametocytes from the overall number tested.

A questionnaire was administered during the malaria survey to determine the association between malaria infection and use of preventive measures. The respondent’s demographic characteristics and use of malaria prevention measures were correlated to their malaria infection status.

### Malaria vector species and transmission potential on Rusinga Island

A longitudinal entomological survey was conducted in Gunda village in Rusinga West sub-location from June to December 2012. Nocturnally active host-seeking mosquitoes were captured and their indoor and outdoor densities and species composition determined. The mosquitoes were collected using odour-baited MM-X traps (American Biophysics, Corp., North Kingston, RI) containing a synthetic mosquito attractant known as Mbita blend [12].

The mosquitoes were collected from six randomly selected village houses spaced at least 25 metres apart [13]. The houses were similar in structure with roofs made of iron sheets, walls made of mud and open eaves. Each house had two rooms. Household members were supplied with untreated mosquito nets that were used throughout the study. Each house was fitted with one MM-X trap per night. The MM-X traps were placed above the foot end of a bed occupied by a human subject. Outdoor biting mosquitoes were collected by hanging traps within the peri-domestic environment approximately 15 cm off the ground. The MM-X traps were operated from 6 pm till 7 am the following morning. Mosquitoes were collected for four nights in a week, with alternating nights for indoor and outdoor trapping. The same houses were used throughout the study. Experiments were replicated over 72 nights, 34 nights with six traps per night indoors (i.e. 204 trap nights) and 38 nights with 6 traps per night outdoors (i.e. 228 trap nights).

Captured mosquitoes were counted, sorted by sex and identified morphologically using the keys of Gillies and DeMeillion [14]. Anopheline mosquitoes were preserved using silica gel awaiting further processing. Legs of female mosquitoes were used to identify the sibling species of *An. gambiae* s.l. using the PCR technique [15]. The head and thorax of female anopheline mosquitoes were tested for the presence of *Plasmodium* sporozoites using an enzyme linked immusorbent assay (ELISA) [16]. The infection rate of mosquitoes captured during the entomological survey was measured as the proportion of mosquitoes found to contain sporozoites.

### Ethical considerations

Ethical clearance was obtained from the Kenya Ethical Review Committee located at the Kenya Medical Research Institute (NON-SSC No. 280). Consent was obtained from participants prior to the malaria survey. Written consent was sought from parents and care givers of children to allow minors to participate in the study. Consent was also obtained from heads of households that provided approval for mosquito collection in houses.

### Statistical analysis

Data were analysed using R statistical software version 2.15.2. Data collected during the malaria survey were analyzed using multivariate logistic regression. The outcome of the malaria test (whether positive or negative for malaria parasites) was treated as the dependent variable. The effect of other variables, specifically sex, location and age group were also analyzed. The response variable in the entomology survey was derived from count data (mosquito numbers). The untransformed data was analyzed by fitting generalized linear models [17] with a poisson regression. The packages mass, effects, epicalc, multcomp, lme4, gee, geepack and aod [18] were loaded before running the analysis. The night mosquitoes were captured (day of experiment) was included in the model as a factor. Statistical significance was set at P < 0.05.

## Results

### Malaria burden and parasite diversity on Rusinga Island

A total of 2,318 individuals from all age groups participated in the malaria survey. The age of study participants ranged from 1 year to 102 years. The majority of the participants were between the age 0 – 14 (n = 1197, 51.7%), while the least were above 60 (n = 131, 5.7%). The median age was 14 years. Of the individuals enrolled in the study 1,263 (54.4%) were female while 1,055 (45.6%) were male (Table 1). Participants from Rusinga East were 1,248 (53.8%) while those from Rusinga West were 1,070 (46.1%).

**Table 1:** **Characteristics of study participants of the survey carried out to determine malaria burden and parasite diversity on Rusinga Island**

**Variable**	**n (%)**
Sex	
Female	1263 (54.4%)
Male	1055 (45.6%)
Age group	
0 – 14	1197 (51.7%)
15 – 30	520 (22.4%)
30 – 45	330 (14.2%)
46 – 60	140 (6%)
Above 60	131 (5.7%)
Median age	14
Location	
Rusinga East	1248 (53.8%)
Rusinga West	1070 (46.1%)
Use of malaria preventive measures	
Yes	2133 (92%)
No	185 (8%)

Overall, blood samples from 252 (10.9%) participants had malaria parasites. Three malaria species were identified namely *Plasmodium falciparum*, *Plasmodium malariae* and *Plasmodium ovale*. Among the 252 individuals harboring malaria parasites 222 (88.1%) were infected with *P. falciparum*, 10 (3.96%) with *P. malariae* and 2 (0.79%) with *P. ovale*. Of the remaining 18 individuals with malaria parasites, 16 (6.34%) had mixed infections of *P. falciparum* and *P. malariae*, 1 (0.39%) had a mixed infection of *P. falciparum* and *P ovale* and 1 (0.39%) had a mixed infection of all 3 malaria species (Table 2). The overall prevalence of mixed *Plasmodium* infections was 7.14%.

**Table 2:** **Malaria infection among individuals from Rusinga East and West locations**

**Location**	**N**	**n**	**Malaria parasite species**					
			*Pf*	*Pm*	*Po*	*Pf + Pm*	*Pf + Po*	*Pf + Pm + Po*
Rusinga East	1248	120	105	6	0	9	0	0
Rusinga West	1070	132	117	4	2	7	1	1
Total	2318	252	222	10	2	16	1	1

N refers to the total number of participants; n refers to the number of individuals positive for *Plasmodium* parasites. *Pf* refers to *Plasmodium falciparum, Pm to Plasmodium malariae* and *Po to Plasmodium ovale.*

Out of the total number of blood smears screened for gametocytes 16 (0.7%) participants tested positive. School-going children had the highest burden of gametocytes. Of the 16 individuals with gametocytes 11 (69%) were 0 – 14 years old, 1 (6.25%) was in the 15 – 30 year age bracket, another 1 (6.25%) in the 30 – 45 year age bracket and 3 (18.7%) were in the 45 – 60 year old age bracket (Table 3). Among the gametocyte carriers 6 (37.5%) were from Rusinga East and 10 (62.5%) from Rusinga West. There was a significant association between malaria parasitemia and location (OR = 1.5, 95% CI 1.14 – 1.97, p = 0.003) (Table 4). Individuals who did not use malaria protective measures whilst sleeping were two times more likely to get malaria (95% CI 1.76 – 3.91, p < 0.001) compared to those who did (Table 4).

**Table 3:** **Proportion of participants with gametocytes in the survey carried out to determine malaria burden and parasite diversity on Rusinga Island**

**Variable**	**N**	**n**
Age group		
0 -14	1197	11 (0.47%)
15 – 30	520	1 (0.04%)
30 – 45	330	1 (0.04%)
45 -60	140	3 (0.14%)
Above 61	131	0 (0%)
Sub-location		
Rusinga East	1248	6 (0.26%)
Rusinga West	1070	10 (0.43%)

N refers to the total number of participants; n refers to the number of individuals positive for gametocytes parasites.

**Table 4:** **Factors associated with malaria parasitemia among residents of Rusinga Island**

**Variable**	**Odds ratio (95% CI)**	**p-value**
Location		
Rusinga East	ref	ref
Rusinga West	1.5 (1.14 – 1.97)	0.003
Malaria preventive measures		
Yes	ref	ref
None	2.65 (1.76 – 3.91)	<0.001

### Malaria vector species and transmission potential on Rusinga Island

A total of 1,681 mosquitoes, including both males and females, were collected (Table 5). The species caught were *An. gambiae* s.l., *An. funestus* s.l., *Culex* species, *Mansonia* species, and *Aedes* species. Among the collected mosquitoes 79 (4.7%; constituting 74 females and 5 males) belonged to the genus *Anopheles* while 1,602 (95.3%; constituting 1,357 females and 245 males) were culicine species. Of the total female anophelines collected, *An. gambiae* s.l. was the most abundant malaria vector (n = 46; 62%). Female *An. funestus* mosquitoes accounted for 38% (n = 28) of the total female anophelines collected. Overall, *Culex* species were the most abundant non-malaria mosquitoes collected (n = 1,493).

**Table 5:** **Number of mosquitoes captured inside (204 trap nights) and outside (228 trap nights) houses in Gunda village on Rusinga Island**

**Species**	**Sum of mosquitoes collected**		**p-value**	**Total**
	**Indoors (%)**	**Outdoors (%)**		
*An. gambiae s.l.* females	19 (41.3)	27 (58.7)	0.477	46
*An. gambiae s.l*. males	3 (60)	2 (40)	0.499	5
*An. funestus s.l.* females	9 (32.1)	19 (67.9)	0.153	28
*An. funestus s.l.* males	0 (0)	0 (0)	1.000	0
*Culex* species females	665 (52.4)	584 (47.6)	<0.001	1249
*Culex* species males	132 (54.1)	112 (45.9)	0.038	244
*Mansonia* spp females	44 (45.4)	53 (54.6)	0.681	97
*Mansonia* species males	0 (0)	0 (0)	1.000	0
*Aedes* species females	7 (63.6)	4 (36.4)	0.291	11
*Aedes* species males	1 (100)	0 (0)	0.997	1
Total	880	801	–	1,681

Although a higher number of female *An. gambiae* s.l. mosquitoes were captured outdoors (n = 27) than indoors (n = 19) these catches did not differ significantly (P = 0.477). Similarly, there was no statistical difference between the number of female *An. funestus* s.l. mosquitoes captured indoors versus outdoors (P = 0.153). Of the 46 *An. gambiae* s.l. mosquitoes subjected to polymerase chain reaction (PCR) analysis, 39 were successfully identified. The rest (7 samples) failed to amplify. Of the 19 *An. gambiae* s.l. females captured indoors, 12 (92.3%) were identified as *An. arabiensis* and 1 (7.7%) as *An. gambiae* s.s. Of the 19 *An. gambiae* s.l. females captured indoors, 6 failed to amplify. Of the 27 *An. gambiae* s.l. mosquitoes captured outdoors, 24 (92.3%) were identified as *An. arabiensis* and 2 (7.7%) as *An. gambiae s.s*. Only 1 specimen of the 27 *An. gambiae* s.l. mosquitoes captured outdoors did not amplify. These data indicate that *An. arabiensis* is the dominant malaria vector among siblings of the *An. gambiae* complex on Rusinga Island.

Of the total 62 malaria vectors tested for the presence of *Plasmodium falciparum* sporozoites 28 and 34 were captured indoors and outdoors, respectively. Overall 4 mosquitoes were sporozoite positive of which 2 (7.14%) were captured indoors (1 *An. arabiensis* and 1 *An. funestus*) and the other 2 (5.88%) outdoors (1 *An. arabiensis* and 1 *An. funestus*). The overall sporozoite infectivity rate was 6.45% (4/62). Site-specific sporozoite rates were 7.14% for indoors and 5.88% for outdoors.

There was a significant difference in the number of *Culex* spp. captured indoors compared to those captured outdoors (P < 0.001). Among the 1,249 female *Culex* species of mosquitoes caught 665 (52.2%) were caught indoors and 585 (46.8%) were collected outdoors. No statistical difference was found between the females of *Mansonia* (P = 0.681) and *Aedes* mosquito species (P = 0.291) captured indoors and outdoors. *Culex* species yielded the largest collection of male mosquitoes (n = 244), with significantly higher numbers of the mosquitoes being collected indoors (n = 132; 54.1%) than outdoors (n = 112; 45.9%) (P = 0.038).

## Discussion

In this study, single infections of *Plasmodium falciparum, P. malariae* and *P. ovale* as well as multiple infections of these species were observed. The overall malaria prevalence on Rusinga Island was 10.9%. The risk of contracting malaria was higher among dwellers of Rusinga West than Rusinga East locations. Parasite positivity was significantly associated with individuals who did not use malaria protective measures. The numbers of females of the main malaria vectors namely *Anopheles gambiae s.l.* (consisting largely of *An. arabiensis*) and *An. funestus* collected indoors and outdoors did not differ significantly. This was also the case with females of species of *Mansonia*. On the contrary significantly more male and female *Culex* species were collected indoors than outdoors. Interestingly the ratios of *An. gambiae* versus *An. arabiensis* in samples collected indoors versus outdoors were similar, *albeit* with overall higher numbers of mosquitoes being collected outdoors. Similarly, the numbers, and more or less the ratios, of malaria infected mosquitoes collected indoors and outdoors were equal.

Malaria prevalence among residents of Rusinga Island was 10.9%. However, a prevalence study conducted on the Island in November 1998 revealed that the prevalence was 24.4% [8]. This suggests that there has been a reduction in the burden of malaria in the study area probably due to the increased use of LLINs [4]. However, the prevalence of malaria reported in this study is generally lower than the 40% reported by Noor et al. around the shores of Lake Victoria in 2009 [1]. Identification of malaria parasites by microscopy largely depends on the skills and experience of the microscopist [19], thus it is likely that the prevalence rate of malaria reported in this study is an underestimate.

Single infections of *P. falciparum, P. malariae* and *P. ovale* as well as multiple infections of these parasites were found in the study area. The predominant malaria species on Rusinga Island was *P. falciparum*. This finding is consistent with those of other studies conducted in Mbita sub-county [8,20,21]. *Plasmodium malariae* was reported as a minor species responsible for malaria infections on Rusinga Island. Previous studies conducted in the area also documented *P. malariae* as being responsible for single and co-infections of malaria in Mbita sub-county [20,21]. In this study, single as well as mixed infections of *Plasmodium ovale* were detected in a few individuals. Reported cases of malaria infections caused by *P. ovale* are rare and could be because of under-diagnosis or low transmission rates [22,23]. The parasite is known for its low densities [24,25] which contribute to difficulties in diagnosis. Furthermore, the greatest shortcoming of the diagnostic technique used in this study i.e. microscopic examination of patients’ peripheral blood smears stained with Giemsa is low sensitivity. This may explain the small number of individuals diagnosed with *P. ovale* malaria in the study area. Cases of *P. ovale* infections have been reported in other areas of western Kenya [26,27].

Malaria burden was found to be unevenly distributed with individuals from Rusinga West location recording more malaria cases. This can be attributed to the higher number of malaria mosquito breeding sites found in Rusinga West compared to Rusinga East (Mukabana, unpublished data). Most of the breeding sites recorded in Rusinga West were man-made [28] and created to sustain livelihood activities. With regards to outdoor fishing activities, more fishing activities are conducted in Rusinga West compared to East. A higher number of fishing beaches engaged in nocturnal fishing activities are found in Rusinga West (five) compared to East (three).

It is not surprising that the gametocyte prevalence detected by microscopy was as low as 0.7%. A study conducted in Mbita hospital, less than two kilometres from Rusinga Island reported a 0.9% prevalence of gametocytes among patients seeking outpatient services [21]. Gametocytes have been known to be low in densities thus can easily go undetected when screened microscopically [29]. Of the total parasite load in an infected person, the proportion of gametocytes has been reported to be 0.2% and 5.7% in children and adults, respectively [30]. Gametocytes are the sexual stages of the malaria parasite responsible for transmission from human to mosquito. This study shows that a small subpopulation of humans is infective and may be responsible for maintaining malaria transmission on Rusinga Island. It is important to note that this infective subpopulation may be higher if the presence of gametocytes is detected using molecular techniques.

This study demonstrated that individuals who did not use malaria protective measures whilst sleeping indoors at night were at a higher risk of malaria. Similar findings have been reported in other parts of Africa [31,32]. The majority of the adult population on Rusinga Island are fishermen who engage in nocturnal outdoor activities and therefore hardly use LLINs consistently. A review conducted by Pulford *et al.* [33] documented several reasons why people do not sleep under LLINs. Spending time elsewhere, for instance, at the work place at night, was cited as a reason for not using an LLIN even when one was available [33]. An increase in outdoor biting has been reported in several areas in sub-Saharan Africa [34-36]. Although approximately 80% of malaria transmission occurs indoors [37], outdoor malaria transmission is still important. Indoor vector control interventions are effective and have been reported to reduce malaria transmission in several areas [38-40], but are insufficient and extra effort is required to eliminate malaria. This should be especially applied in settings with intense regular nocturnal outdoor human activities. Since malaria transmission occurs where mosquitoes bite humans, vector control strategies need to be developed that also target outdoor biting mosquito populations [41] in order to achieve malaria elimination [42-45].

The lack of a statistical difference between the numbers of female malaria vectors collected indoors and outdoors, with higher numbers of the mosquitoes being collected outdoors than indoors, underscores the epidemiological importance of understanding and investing in outdoor transmission control in the current big push towards eliminating malaria [46]. It is likely that *An. arabiensis* and *An. funestus* are responsible for transmitting malaria indoors and outdoors in the study area. *Anopheles arabiensis* is known to be opportunistic, preferring to feed outdoors [47] on humans and animals depending on availability [48]. Several studies have also reported *An. arabiensis* as being partially responsible for indoor malaria transmission in several areas [49-52]. *Anopheles funestus* s.s. Giles has been identified as the main vector among siblings in the *An. funestus* group found in Mbita sub-county [4]. This vector prefers to rest indoors and feed on humans [53,54].

The findings of this study corroborate those of Futami *et al*. [4] who reported that *An. arabiensis* had replaced *An. gambiae* as the main malaria vector in the study area. Similar cases of species shifts among populations of malaria vectors have been reported elsewhere in Kenya [3,55,56] and Tanzania [57]. The species shifts among *An. gambiae* and *An. arabiensis* populations were reported to occur after wide coverage, ownership, and use of LLINs [3,55]. *Anopheles arabiensis* has been known to survive in areas with wide coverage of both LLINs and Indoor Residual Spraying (IRS) [5,34,56]. Killeen [46] indicated that *An. arabiensis* mosquitoes have adopted a behaviour that is instrumental in avoiding prolonged exposure to insecticides. These malaria vectors exit houses immediately after entry if a human host is sleeping under an LLIN [46], suggesting that the mosquito either enters another house or searches for a blood meal host outdoors. This particular behaviour has been reported in several studies [58-60].

Interestingly the ratios of *An. arabiensis* versus *An. funestus* in samples collected indoors versus outdoors were similar. This touches on the lack of bias of the sampling tool for indoor and outdoor collections. A possible explanation is that since outdoor collections were done on the outer walls of houses, we were dealing with the same mosquito subpopulations. The finding that no significant difference was found in density of *An. arabiensis* and *An. funestus* found indoors and outdoors is most likely explained by the few number of mosquitoes captured. It is statistically difficult to show differences in indoor and outdoor mosquito densities when general mosquito densities are low. It is unlikely that the low density of anopheline mosquitoes captured on Rusinga Island reflects a lack of efficacy of the MM-X trap as a sampling tool. The trap has been successfully used in sampling wild mosquitoes in sub-Saharan Africa [12,61-63]. More specifically, MM-X traps baited with the Mbita blend (as used in this study) have been shown to trap high numbers of *Anopheles* mosquitoes in other areas of western Kenya [12]. Also worth noting is that although there were few anophelines the numbers of culicine mosquitoes trapped in this study were high, indicating good sampling efficacy of the MM-X traps.

High densities of *Culex* mosquito species were recorded in this study. Other non-malaria vectors captured included *Mansonia* and *Aedes* spp. The presence of these mosquitoes indicates the potential transmission of arboviruses in Mbita sub-county. However, arboviral diseases have not been diagnosed in health facilities in Mbita sub-county, probably due to lack of diagnostic tools. We posit that the aforementioned non-malaria vectors are involved in nuisance biting in the area. One point is that targeting *Anopheles* species and leaving out culicine species may reduce acceptance of malaria control interventions among target communities because of the continuing biting menace from the unaffected *Culex* population [64-66].

It was interesting to note that the numbers and more or less the ratios, of malaria infected mosquitoes collected indoors and outdoors were equal. Malaria mosquitoes mainly bite at night when humans are asleep [47]. Thus, individuals who use indoor mosquito control tools are presumably protected from malaria infective bites. The front-line malaria vector control interventions specifically IRS and LLINs are effective indoors, which is a major space for insecticidal exposure [37]. These indoor interventions do not protect individuals who spend a significant part of their time outdoors and at night. This is the case of the capture fishing community of Rusinga Island where, in addition, the local malaria vectors prefer to blood-feed at night [4,47,67-69]. Huho and others [37] indicate that human behaviour is an important determinant in the place where malaria transmission occurs and it is strongly argued that outdoor transmission persists in areas with intense nocturnal outdoor activities [46,70]. The convergence between outdoor nocturnal fishing activities, preference for night-biting by local malaria vectors and the similarity in sporozoite rates between malaria mosquitoes collected indoors and outdoors exemplifies the vicious cycling of the disease in rural Africa.

Malaria mosquitoes on Rusinga Island were found to be low in density. This may be explained by climatic conditions, particularly fluctuating rainfall intensity experienced in recent years in the study area. The low adult mosquito density has previously been reported by Futami *et al*. [4] who compared densities of *An. gambiae* s.l. females in selected years from 1999 to 2010. Futami and others [4] reported a 95% decline in densities of *An. gambiae* s.l. which was attributed to an increase in bednet coverage. Evidence collected in the study reported herein implies that a small population of malaria vectors is responsible for malaria cases on Rusinga Island. Small populations of malaria vectors can sustain high malaria transmission in endemic areas [71]. Low densities of malaria vectors also imply that malaria vectors on Rusinga Island are highly efficient and maintain stable malaria transmission throughout the year. Elimination of malaria from Rusinga Island will require an increase in the use of interventions that target indoor transmission as well as novel interventions to control outdoor transmission.

## Conclusion

The rather significant and unexpected contribution of *P. malariae* and *P. ovale* to the overall malaria prevalence on Rusinga Island underscores the epidemiological importance of these species in the big push towards eliminating malaria. Although current entomological interventions mainly target indoor environments, additional strategies should be invented to prevent outdoor transmission of malaria.

## Abbreviations

CI: Confidence interval
CSA: circumsporozoite antibodies
ELISA: Enzyme-linked immunosorbent assay
HDSS: Health Demographic Surveillance System
IRS: Indoor residual spraying
MM-X: Mosquito magnet-x trap
PCR: Polymerase chain reaction
OR: Odds ratio
